# Draft genome sequence of *Teratoramularia rumicicola* strain TR4 isolated from *Rumex crispus*

**DOI:** 10.1128/mra.01309-25

**Published:** 2026-02-13

**Authors:** Masataka Izumi, Tomomasa Oka, Toyozo Sato

**Affiliations:** 1 Graduate School of Agriculture, Kyoto University 12918, Kyoto, Japan; 2 National Institute of Global Health and Medicine, Japan Institute for Health Security 739298 , Shinjuku-ku, Tokyo, Japan; 3 Niigata Agro-Food University, Tainai-shi, Niigata, Japan; Rochester Institute of Technology, Rochester, New York, USA

**Keywords:** *Teratoramularia rumicicola*, bioherbicide, Dothideomycetes, *Ramularia*-like fungi, draft genome sequence

## Abstract

We present the draft genome sequence of *Teratoramularia rumicicola* strain TR4, a potential bioherbicide isolated from *Rumex crispus* and sequenced using the DNBSEQ technology. This genome provides a foundational resource for future studies on taxonomy, pathogenicity, and secondary metabolite biosynthesis in *T. rumicicola*.

## ANNOUNCEMENT

There are currently no genome assemblies available for the genus *Teratoramularia* in public repositories, including *Teratoramularia rumicicola*. A genome sequence for *T. rumicicola* provides a genomic reference that can support future taxonomic and comparative studies of *Ramularia*-like fungi, as well as research on pathogenicity and the potential use of this species as a microbial herbicide. *Teratoramularia rumicicola* TR4 was isolated by single-conidium isolation from a naturally infected *Rumex crispus* leaf in Tsukuba, Ibaraki, Japan in November 2024 and deposited as MAFF 248116 (1). Species identification based on morphology and ITS/LSU phylogeny has been described previously (1). Here, we focus solely on genome generation and assembly metrics.

*Teratoramularia rumicicola* strain TR4 was grown on potato dextrose agar (PDA) at 25°C for 23 days in the dark. Mycelium was scraped from PDA plates, transferred to cryovials, and stored at −80°C until genomic DNA extraction. Genomic DNA was extracted from mycelium by Bioengineering Lab. Co., Ltd. using the MPure Bacterial DNA Extraction Kit (MP Biomedicals, Santa Ana, CA, USA) on an MPure-12 system following the manufacturer’s protocol. Sequencing libraries were prepared with the MGIEasy Fast FS Library Prep Set v2.0 (MGI Tech, Shenzhen, China), circularized, converted into DNA nanoballs, and sequenced on a DNBSEQ-T7 instrument in 2 × 150 bp mode. The run yielded 20,667,958 paired-end reads (6.20 Gb). Adapters were removed, and reads were quality-filtered with fastp v0.20.1 (2) using MGI adapter sequences and otherwise default parameters. All bioinformatic analyses were performed using default parameters, except where otherwise noted. The sequencing data yielded an average coverage of approximately 290×. The filtered reads were assembled *de novo* with SPAdes v4.2.0 (3), and the draft assembly was polished once with Pilon v1.24 (4).

After removing one contig that showed markedly elevated coverage and was identified as mitochondrial by BLASTn, the final nuclear genome assembly contained 541 contigs in total, of which 316 contigs (≥500 bp) totaled 21.32 Mb (guanine or cytosine [GC] content 55.08%), with an N50 of 0.284 Mb, a largest contig of 0.663 Mb, and an L50 of 25, as computed with QUAST v5.3.0 (5). Genome completeness assessed with BUSCO v6.0.0 (dothideomycetes_odb10) was 96.5% complete (single-copy 96.4%, duplicated 0.1%), with 0.2% fragmented and 3.3% missing (6).

## Data Availability

This Whole Genome Shotgun project has been deposited at DDBJ/ENA/GenBank under accession JBSLQR000000000 (BioProject: PRJNA1367340; BioSample: SAMN53337873). The version described in this paper is version JBSLQR010000000. Raw sequencing reads are available in DRA under accession DRR802130.
